# HIV Digital Vaccine Strategy: Proposal for Applying Blockchain in Preventing the Spread of HIV

**DOI:** 10.2196/37133

**Published:** 2022-06-13

**Authors:** Jia Liu

**Affiliations:** 1 Henan Center for Disease Control and Prevention Zhengzhou China

**Keywords:** HIV, blockchain, digital vaccine, decentralized surveillance

## Abstract

**Background:**

The HIV epidemic imposes a heavy burden on societal development. Protection of susceptible populations is the most feasible method for eliminating the spread of HIV. In the absence of a biological vaccine, the definitive solution is enabling susceptible populations to recognize and avoid high-risk sexual behavior.

**Objective:**

The objective of this study is to use specific technologies and strategies to establish a system by which high-HIV-risk individuals can determine the HIV infection status of one another anonymously, conveniently, and credibly.

**Methods:**

This study proposes an HIV digital vaccine (HDV) strategy, a decentralized application (Dapp) based on blockchain for use by individuals with a high risk of HIV and accredited testing agencies (ATAs). Following testing, only the HIV-negative results (or linked information) are uploaded to the blockchain, which results in high-risk individuals being able to determine the HIV-negative status of each other anonymously, conveniently, and credibly.

**Results:**

Future work includes the following: (1) a survey of the willingness to use Dapps among high-HIV-risk populations, (2) a larger framework containing both HDV and people living with HIV (PLH) and discussing the influence of HDV on PLH and its possible solutions, and (3) coordinating with the blockchain development team, ATAs, community-based organizations, and third-party organizations to raise funds, develop the Dapp, formulate detailed plans, and publicize and promote it. The exact timeline for achieving these objectives cannot be determined at present.

**Conclusions:**

The HDV strategy may reduce the occurrence of high-risk sexual behavior and effectively protect susceptible populations; combined with current strategies, it is a promising solution to prevent the spread of HIV. The included concepts of decentralized surveillance and surveillance as intervention may spark a change in current infectious disease prevention and control modes to introduce beneficial innovations in public health systems globally.

**International Registered Report Identifier (IRRID):**

PRR1-10.2196/37133

## Introduction

### Background

The HIV epidemic is a major global public health problem. Between 2000 and 2015, the international community provided approximately US $109.8 billion worth of development assistance for HIV prevention and control [1]; however, the spread of HIV has not been effectively curtailed, with approximately 2 million new HIV infections occurring globally every year [2]. At the 20th International AIDS Conference in 2014, the Joint United Nations Program on HIV and AIDS adopted the 90-90-90 strategy to help end the HIV epidemic. By 2020, 90% of all people living with HIV (PLH) should know their HIV status, 90% of all people diagnosed with HIV infection should receive sustained antiretroviral therapy, and 90% of all people receiving antiretroviral therapy should achieve viral suppression [3]. The core components of this strategy require substantial scaling up of HIV testing to identify sources of infection, subsequent scaling up of treatment, and assurance of effective treatment to control the sources of infection. However, this strategy requires a huge amount of resources [4], and researchers have expressed different opinions on its feasibility [5-7]. Although many countries and regions have made good progress, the United Nations AIDS report shows that in 2020, the global target of 90-90-90 has not been met, and a large gap remains in most countries and regions. At the same time, low- and middle-income countries are 30% short of the funding needed to meet the 90-90-90 targets, and this gap is widening [8]. It is apparent from the lack of cost considerations that the 90-90-90 strategy is the best attempt by the international community in the absence of a vaccine.

### Main Problems Faced in Preventing the Spread of HIV

Considering the chain of infection (infection source, transmission route, and susceptible population), it is apparent that implementing preventive measures for all sources of infection is extremely difficult. In a previous study, based on a mathematical model, the estimated cost per new HIV case diagnosed in the United States was at least US $2528 and could be up to US $63,053 under specific circumstances [9]. Because of factors including incurability and the long asymptomatic stage of HIV as well as the protection of individual rights, even if most sources of infection are found, it is impossible to control them thoroughly and effectively. Furthermore, the management of HIV transmission routes, which are mainly sexual, cannot be realized because sexual activity is a basic human need. Therefore, currently, the most feasible method for preventing HIV spread is protecting susceptible populations. In the absence of a vaccine, the definitive solution is enabling susceptible populations to recognize and avoid high-risk sexual behavior. However, the key barrier to this method is the lack of a system by which individuals can determine the HIV infection status of one another conveniently, anonymously, and credibly. Therefore, the use of specific technologies and strategies to establish such a system has great significance for protecting susceptible populations against HIV infections.

### Attempts at Solving the Problem

To solve this problem, the HIV infection status of individuals must first be known. Forced by circumstances [10], the Chinese government used its administrative power to launch a real-name system for universal HIV screening of former paid blood donors in 2004 [11]. Subsequently, comments regarding HIV testing and the issue of individual rights were raised in an article [12]; this approach had many disadvantages that outweighed the benefits of social balance. Therefore, in noncritical situations, the government may not be inclined to use such methods. More importantly, without effective control of the infection sources and transmission routes, HIV spread cannot be eliminated.

The advent of the internet has changed the strategies of disease prevention and control [13]. With the popularization and convenience of the internet, an increasing number of studies have used this medium for the testing, intervention, and management of specific diseases or for providing relevant services [14-16]. The feasibility, acceptability, and influence of novel internet technologies in HIV infection prevention have also been confirmed [17,18]. Current internet-based testing, management, intervention, and treatment methods aim to identify HIV-infected persons and provide treatment and behavioral intervention. Though it is helpful to approach 90-90-90 targets, this strategy is unable to address the need for a method that allows individuals to check the HIV infection status of one another conveniently, anonymously, and credibly.

### Overview of Blockchain Technology and the Surrounding Concept

The advent of blockchain technology offers possibilities for solving the aforementioned problems. Blockchain technology is the underlying foundation of cryptocurrencies such as Bitcoin and was first described in the foundational article entitled “Bitcoin: A Peer-to-Peer Electronic Cash System” published in 2008 [19]. China’s Ministry of Industry and Information Technology defined blockchain technology as a novel application of computer technologies, including decentralized data storage, peer-to-peer transfers, consensus mechanisms, and encryption algorithms, in the internet era [20]. With blockchain technology, information is encrypted and anonymously recorded in a network. As the recorded information is indelible, open, and transparent, a highly effective and convenient decentralized network storage system can be established. These properties help society achieve lower costs of trust, enable wider collaboration, and provide the foundation for a form of self-organization independent of governments and markets [21,22]. With the industry changing constantly in recent years, application-based research in the fields of health and medicine is also increasing continuously [23-25].

Smart contracts add more value to the blockchain ecosystem. They are executable codes that run on top of the blockchain to facilitate, execute, and enforce an agreement between untrustworthy parties without the involvement of a trusted third party [26]. The decentralization, autoenforcing ability, and verifiability of smart contracts enable their encoded business rules to be executed in a peer-to-peer network, where each node is “equal,” and none has any special authority without the involvement of a trusted authority or a central server. Smart contracts are expected to revolutionize many traditional industries, such as finance, health care, and energy [27].

Smart contracts can be used as standalone applications. For instance, smart contracts are commonly used to create a tradeable digital token, which can represent a currency, an asset, a virtual share, or a proof of membership. A smart contract may define a token with a fixed supply or even act as a central bank that can issue tokens [28]. Tokens are created on top of existing blockchains, and cryptocurrency is native to its own blockchain. In practice, cryptocurrencies and tokens are frequently used interchangeably. Here, crypto asset is used as an umbrella term. Crypto assets represented by Bitcoin are an important element of blockchain applications. A token represents the typical crypto asset; its simple issuance, transparent distribution, efficient circulation, and programmable characteristics are all advantages over the existing banking and currency system [26]. The improvement and abundance of tokens make communication between various entities cheaper and collaboration more efficient, thus making the application of blockchain possible in more scenarios.

Alternatively, a smart contract may be used as the back end of a multitiered application. Any application that relies on one or more smart contracts as its back end is known as a decentralized application (Dapp) [28]. In contrast to traditional applications in which the back-end code runs on centralized servers, Dapp is a novel form of the blockchain-empowered software system [29]. Its main properties include being open source, providing internal cryptocurrency support, ensuring decentralized consensus, and having no central point of failure [30]. In practice, an ideal Dapp should be completely hosted by a peer-to-peer blockchain system and will need no maintenance and governance from the original developers. The cost and profit are shared by all participants in the decentralized autonomous organization [31]. Currently, owing to the performance limitation of the current blockchain and smaller ecosystem, smart contracts still need to be run locally to complete the application [29]. Nevertheless, many Dapps have already been running stably and effectively on smartphones. The latest reports have shown 1 Dapp’s daily user count reaching 1 million users [32]. Crypto assets are used to purchase services in Dapps. Information and assets of users are interacted with and exchanged via Dapps. As the number of users increases and the application mode expands, webs of trust with different scopes can be formed among people.

The objective of this study was to use blockchain and its ecosystem to establish a system by which high-HIV-risk individuals can determine the HIV infection status of one another conveniently, anonymously, and credibly.

## Methods

### HIV Digital Vaccine (HDV) Strategy and Decentralized Surveillance

**Figure 1:**
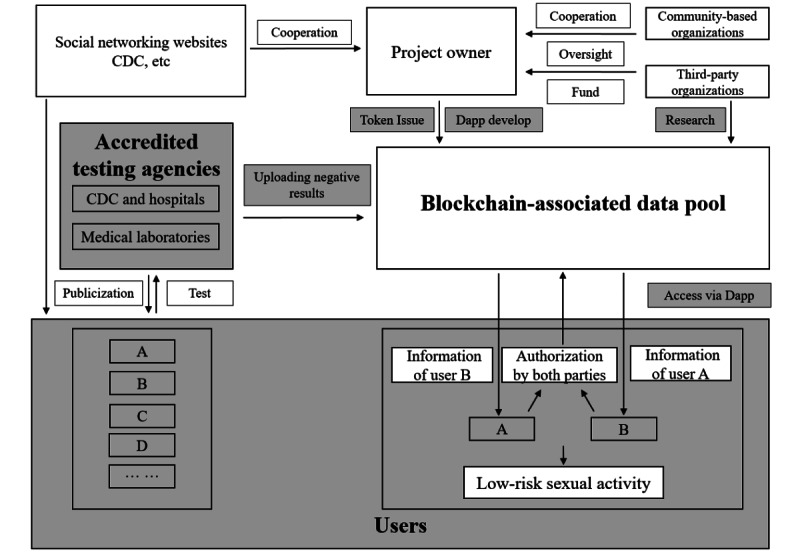
Technical flow chart of the HIV digital vaccine strategy. A, B, C, and D represent individuals belonging to high-HIV-risk populations. Tokens are exchanged when transactions occur among participants. Various transaction patterns can be used in different regions and they may vary when the situation changes. Behaviors in the gray frames are associated with blockchain. The participants in the gray frames are the major users of the Dapp. CDC: Centers for Disease Control and Prevention; Dapp: decentralized application.

Under the oversight of community-based organizations, the project owner develops a Dapp based on the public permissionless blockchain. Subsequently, the Dapp is publicized to facilitate the addition of individuals belonging to high-HIV-risk populations as users. After testing is completed at accredited testing agencies (ATAs), only the HIV-negative results (or linked information) are uploaded to the blockchain. Fingerprint recognition is used to ensure the reliability of the user's identity in the case of anonymity, and the parties involved in high-risk activities can access the blockchain via their smartphones to check the HIV-negative status of one another conveniently, anonymously, and credibly. Thus, high-risk populations can avoid high-risk sexual behavior and gain effective protection against HIV infections. As the number of users increases, prevention of HIV spread can be ultimately achieved. Figure 1 shows the technical flow chart of the strategy.

### Segments of the HDV strategy

The main segments of the HDV strategy are as follows:

1. High-HIV-risk populations benefit the most from the prevention of HIV spread, and therefore have the motivation to promote this strategy. Men who have sex with men (MSM) constitute one of the key populations in the global HIV epidemic scenario [3]; more importantly, they have their own communities, especially internet-based communities such as Jack'd and Blued, which have the largest concentration of MSM in the United States and China, respectively [33,34]. For these reasons, MSM-specific social software operation teams (which often include MSM) are a natural fit for the project. Hospitals, as primary ATAs with closely related interests and the ability to provide more health services, are also suitable as joint project owners. It is ideal for the operation team and hospitals to jointly become project owners, which is more convenient for the collaboration between users and service providers. In view of the public interest characteristics of the strategy, the public permissionless blockchain platform is the best choice.
2. Dapp comprises two types of users: general users and ATAs. ATAs are genuine and authentic agencies (eg, Center for Disease Control and Prevention, hospitals, and medical laboratories) that can conduct actual tests and upload the test results of general users. ATAs post test results directly on the blockchain via Dapp or regular wallets (only with the time-stamped test result). General users will then have approved the addition of such data to the chain attached to their blockchain address. After the test results (or linked information) are uploaded to the blockchain, general users can authorize other users to access relevant test results. In some cases, to avoid using a Dapp, which is limited and not very user-friendly, a regular mobile app linked to an off-chain data repository that connects to the actual blockchain via smart contracts could be another option (private key management via fingerprint). Importantly, no general user registration is required, and no personal information is collected. Thus, the data on the server are totally anonymized.
3. Publicization of the Dapp is an important segment of this strategy. Maintaining complete anonymity for general users is the basis for publicization and promotion. Initial publicization efforts must be focused on populations with a strong demand through ATAs, community-based organizations, social networking websites, and HIV publicity activities; tokens could be used as incentives both for these entities and users. Further publicization can be performed among other high-risk populations after accumulation of experience and results.
4. A token used in the strategy mainly for information exchanges and value transfers, which can be the native cryptocurrency or the already-issued stable coin on the special public-permissioned blockchain, can meet the need. Furthermore, if extension of the strategy is considered, a larger ecosystem may lead the project owner to consider issuing a new token for crowdfunding and coordination of interests. These extensions may include addressing the negative externalities of the strategy (ie, compensating for the potential impact on PLH), other health services for high-HIV-risk populations, or prevention and control of other sexually transmitted diseases. Such a larger ecosystem cannot rule out the possibility of forming a new public chain.
5. Tokens can be issued by the project owner and used in transactions in the Dapp, which include the following: (1) A portion of the tokens is initially distributed to participating entities. (2) Project owners can promote the use of the Dapp by rewarding users with tokens. (3) Tokens are exchanged among users to allow for checking test results. (4) Users can use tokens to purchase additional Dapp services. (5) The government and industry can use data in the blockchain for monitoring, assessment, and research purposes. Following routine assessments, the government can purchase tokens in exchange for acquired social benefits. Various requirements ensure the circulation of tokens. Multiparty transactions of tokens ensure that the token economy system forms a closed loop for value realization, whereas listing and circulation provide a market safeguard for the token value.
6. When the segments described above are realized, the participating parties, which mainly comprise high-HIV-risk populations, jointly form a self-organizing community that can perform real-time information exchanges and value transfers.

The settings for the project owner, Dapp, and token are open and flexible. More possibilities exist as new models/infrastructures emerge. For example, the non-fungible token approach, which has recently gained much attention [35], is also worth considering. Users can grant access to each other's test reports by sending non-fungible tokens.

The voluntary checking of HIV status by individuals falls under virtual surveillance, which occurs before high-risk sexual behavior; both the implementers and recipients are HIV-negative individuals. This differs from the current mode of surveillance conducted by governments and industry, and this new mode of surveillance can be named decentralized surveillance.

In the decentralized surveillance network, which is jointly constructed by involving individuals, people can check the HIV-negative status of each other conveniently, anonymously, and credibly. Provided that 1 user is unable to confirm the HIV-negative status of his temporary sexual partners, he would refuse to have sex or take advantage of other interventions (such as condoms or pre-exposure prophylaxis) to protect himself, thereby providing the user with the ability to avoid high-risk sexual behavior and preventing HIV infections. As a matter of fact, during the process of surveillance, real-time intervention occurs automatically. The seamless conjunction mode of surveillance and intervention is indeed surveillance as intervention (SasI); the publicization and use of SasI in populations of an adequate scale can effectively curb new HIV infections. As this blockchain technology–based strategy is similar to the vaccination strategy and the effects of its application are identical to those of biological vaccines, this proposed strategy has been named the HDV strategy.

## Results

This study aims to propose and discuss the concept and framework of HDV, with a considerable amount of future work to be conducted:

1. A survey of the willingness to use Dapps among high-HIV-risk populations is needed to provide a basis for formulating the scheme to implement the HDV strategy. The survey is being prepared for use in Zhengzhou, China, and the survey protocol is currently being refined.
2. A larger framework containing both HDV and PLH is needed, accompanied by analysis of the influence of HDV on PLH and its possible solutions. Research on financial incentive frameworks for HIV treatment is being conducted; the payments made by high-HIV-risk populations in the HDV strategy can be used as financial incentives for HIV treatment populations, provided that the financial incentives for HIV treatment are reasonable, precise, and efficient. The HDV incentive for HIV treatment and HIV treatment back to HIV prevention (effective treatment suppresses viral load and prevents HIV transmission) forms a good ecology of HIV prevention and treatment, and it helps reduce the social costs of HIV containment.
3. Coordination with the blockchain development team, ATAs, community-based organizations, and third-party organizations (charities, investment institutions, research institutes, etc) is necessary to raise funds, develop the Dapp, formulate detailed plans, and publicize and promote the HDV strategy. The exact timeline for these objectives cannot be determined at this time.

## Discussion

### Significance of the Study

This study proposes the blockchain-based HDV strategy to enable individuals to avoid high-risk sexual behavior and thereby prevent new infections in the population level. The new public health concepts and models such as HDV, decentralized surveillance, and SasI were introduced for the first time. A significant amount of groundwork is required before HDV can be implemented, as mentioned in the Results section. As blockchain brings HIV prevention to a completely new field, the necessity, feasibility, and challenges merit further discussion.

### Necessity of Decentralization and Advantages of Blockchain

We consider whether non-blockchain–based centralized methods can enable the verification of HIV infection status conveniently, anonymously, and credibly. The answer to this question can be derived from simple reasoning. The strategy in question is relatively simple and would have been previously considered or implemented by the government, industry, or even community-based organizations. However, the fact that this strategy never gained traction demonstrates the existence of certain unassailable barriers. These barriers involve multiple political, economic, social, and psychological concepts and may include, but are not limited to, the following interlinked problems:

1. Government direction (or entrusted to a third party) is the conventional approach under the current system. The HDV strategy may lead to the isolation of HIV-infected people, whereas government direction may result in a higher tendency for HIV-infected people to experience a strong sense of exclusion.
2. The HDV strategy involves multiple types of entities (such as governments, hospitals, medical laboratories, community-based organizations, and social networking websites), and it is difficult to coordinate their interests. The existing government/business direction approach is inefficient, thus resulting in a low cost-benefit ratio and making long-term implementation impossible.
3. In general, credibility and anonymity cannot be concurrently achieved with centralization, leading to low acceptability in high-risk populations.
4. Centralized servers and real-name systems are at risk of data breaches, with the risk increasing with the scale of publicization.
5. The high costs of collaboration between countries/regions restrict the entire strategy once a single problem becomes irresoluble and results in apprehension from governments, whereas low acceptability among users also poses a barrier to sustained publicization. Therefore, non-blockchain-based centralized methods cannot provide an effective means for checking HIV infection status among individuals conveniently, anonymously, and credibly.

In view of the aforementioned problems associated with centralized strategies, the following advantages of the blockchain are apparent:

1. The property of decentralization makes blockchain technology naturally suitable for approaches not requiring government direction. Individuals are free to choose whether to use Dapp, and the absence of government direction leads to a weaker sense of exclusion.
2. Blockchain brings new forms of self-organization. The issuance and distribution of tokens facilitates consensus among participating entities, allowing for efficient and effective collaboration. Moreover, trading and circulation of tokens facilitates the flow of information and value between participating entities and users, which is a unique advantage of blockchain.
3. Anonymity and credibility are the basic characteristics of blockchain. Dapp does not require the user's basic information.
4. Decentralized information storage will prevent large-scale data breaches, and information leakage in systems without real names does not have a significant effect on individuals.
5. The blockchain-enabled efficient approach reduces collaboration costs between countries/regions.

Certainly, in extreme cases (such as a high prevalence of HIV or where there is a trend in a certain region), the government may use a similar strategy without blockchain by expending significant amounts of resources to mandate screening and registration, but such coercion may be costly, disruptive to liberty, and eventually difficult to implement. Therefore, from a political economy viewpoint, the HDV is feasible simply because of the lower organizational and transaction costs and minimal interference with individual liberty enabled by the blockchain.

### Feasibility

The HDV is a simple strategy. Testing facilities, community-based organizations, and social networking websites can establish a complete system and achieve effective system operation by integrating existing resources. The use of sexual and health needs as motivators for the acceptance of Dapp by high-risk populations is in line with human nature, and the concept that testing facilities and social networking websites are rewarded by the prospect of occupying the most advantageous position on the blockchain is in line with the long-term interests of such organizations. The acquired data can be used in public health services to benefit testing facilities and community-based organizations, ultimately contributing to the overall benefit of humankind. In addition, with tokens as a continuous reward system, this strategy falls in line with the principal benefits of the various participating entities; therefore, there is ample driving force to guarantee the feasibility of the strategy.

Blockchain technology can guarantee that the information in the network is immutable, but it cannot solve trust issues in the real world. With respect to the HDV, the main problems are ensuring that the results can only be used by their owners and preventing malicious use by other parties. Fingerprint recognition technology is widely used in smartphones. If Dapp is set to use only fingerprints as the unlocking method, then the entire process, including account generation, test and result uploading, result display, and transaction, would only use a unique and an unchangeable fingerprint. This would solve the malicious usage problem.

The cost-benefit ratio is an important factor that determines whether a strategy can be implemented. As this strategy is aimed at curbing HIV spread, its cost-benefit ratio should be compared with the total costs of HIV/AIDS prevention in the current society. Currently, government efforts in terms of publicity, testing, and services are ongoing; therefore, the integration of publicization and testing into the strategy does not incur additional costs, whereas the development and maintenance of Dapp only requires minimal human and financial resources. Part of the token value may have to be realized by the government through the purchase of acquired social benefits; however, such a move is relatively simple and cheap compared to launching a comprehensive HIV/AIDS prevention program. In addition, the huge social benefits arising from implementing this strategy are inestimable, thereby resulting in an extremely high cost-benefit ratio when combined with the relatively lower costs.

### Innovations and Outlook

Taking the United States as an example, current HIV surveillance collects, analyzes, and disseminates information about new and existing cases of HIV infection through the National HIV Surveillance System. By meeting the surveillance goal, the Center for Disease Control and Prevention can direct HIV prevention funding to where it is needed most [36]. After allocating funds, the government uses numerous proven interventions to prevent new HIV transmissions [37]. The entire process from initiation of surveillance to implementation of interventions is complex, time-consuming, and costly, but this is indeed the status quo of HIV surveillance and intervention in most countries. Different from the current surveillance process, HIV infections are not considered in decentralized surveillance, and the data are no longer required by the government for collection, processing, and deciding how to implement interventions. Instead, the data are collected by users and immediately used for self-intervention, namely the SasI. Methods that ensure convenience and anonymity, data flows that are fast and secure, and a convenient and flexible token reward system enable the joint participation of individuals with relevant demands. Data on infectious diseases can be collected and shared, thus concurrently enabling self-protection and the prevention and control of infectious diseases. Hence, the SasI mode is more precise and efficient. With the development of blockchain, such a model may potentially spark the change of current government-funded infectious disease prevention and control modes and can bring about beneficial innovations in public health systems globally.

A vaccine is derived from Variolae vaccinae, which was first developed by Edward Jenner from cowpox. The term was created during the first industrial revolution by Louis Pasteur to honor Jenner and cover the new protective inoculations. It has since been expanded to denote a biological preparation that provides active acquired immunity to a particular disease. Benefiting from the information technical revolution, the concept of a vaccine has been further expanded in this paper. According to the discussion of the HDV, we can portray the concept of a digital vaccine. It is a strategy based on new digital information technology for establishing effective channels for information communication and value exchange among target populations so that individuals are capable of avoiding particular infection sources. With the wide application of blockchain in health care, the concept of a digital vaccine could be extended and improved, expanding the concept of a vaccine.

The HDV can provide a more efficient and thorough solution for the protection of susceptible populations; combining it with the present HIV prevention strategies directed by “90-90-90” could serve as a promising solution to prevent HIV spread.

### Challenges and Responses

This strategy is for prevention and does not bring direct benefits to PLH. Because a mutual check of HIV infection status is needed before sexual behavior among high-HIV-risk individuals, who access protection from the HDV, it may be unpleasant to check for HIV infections even if they are not involved. Privacy should be fully protected and free of discrimination. Meanwhile, the rights of HIV-susceptible populations against uninformed infection should also be ensured. HIV- positive individuals are obliged to inform their infection status to sexual partners, but this cannot be fully ensured only through moral restraint. This strategy does not impose any restrictions on HIV-positive individuals and can be applied to protect susceptible populations from uninformed infection. It can balance the rights of various individuals. From a broader perspective, when the health of the entire population is guaranteed, humans have enough resources to cure diseases including AIDS, which will eventually benefit HIV-positive individuals. Preventing the transmission of HIV requires much more resources, which could indirectly slow the progress of studies on curing AIDS. Measures such as devoting a proportion of tokens paid by users to the research on treatment and cure of AIDS should be considered for practical implementation.

In particular, because there exists a window period and test results visible in the Dapp are uploaded 15 days ago, users with HIV-negative results may have been contaminated with HIV or become HIV positive later. The issue of the discordance between the actual status and test results still carries the risk of leading to HIV transmission, but it can be solved through the setting of the Dapp usage rules; the analysis and resolution are presented in Multimedia Appendix 1.

If the tokens of the HDV ecosystem are newly issued, the possibility of tracking wallet owners that own tokens to identify them as high-HIV-risk individuals exists, provided that users exchange real-world resources for the tokens. Solutions include the project owners/ATAs providing services that exchange the token to fiat currency or mainstream stable coin/cryptocurrency or a service for users with privacy needs for converting the token to other anonymous cryptocurrencies on exchanging. As long as the HDV ecosystem continues to evolve, users’ needs will always be met gradually.

The emergence of blockchain technology and its applications in interdisciplinary fields are in the early stages. The transaction speed and volume of clinical data, privacy and security, patient engagement, and incentives are major barriers in the health care field [38]. Meanwhile, there is no prior similar study on the HDV strategy that can be used as a reference or a standard academic system that could provide scientific support; some arguments concerning the strategy have been deduced based on the development logics of human nature, and thus unpredictable issues may emerge during the actual application. Therefore, this study proposes the concept and framework of the HDV strategy, although not as a detailed practice program, which needs to be jointly explored and developed by practitioners in multiple industries.

## Appendix

Multimedia Appendix 1 Analysis and resolution of the discordance between user test results and actual status in HIV digital vaccine strategy.

## Abbreviations

ATA: accredited testing agency
Dapp: decentralized application
HDV: HIV digital vaccine
MSM: men who have sex with men
PLH: people living with HIV
SasI: surveillance as intervention
