# Supplementary material for: HIV Digital Vaccine Strategy: Proposal for Applying Blockchain in Preventing the Spread of HIV
Source: JMIR Res Protoc. 2022 Jun 13;11(6):e37133. doi: 10.2196/37133 (PMC9237779; doi:10.2196/37133)
Supplement: Multimedia Appendix 1 [file resprot_v11i6e37133_app1.doc]

**Supplementary 1**

**Analysis and resolution of the discordance between user test results and actual status in HDV**

In HDV strategy, users with HIV-negative results may actually have being contaminated with HIV or being HIV-infected later. We refer to these individuals the people with expired and false result (PEFRs), they arise from the following two conditions: 1. Window period: the time period from exposure to HIV infection to when the body produces enough HIV antibodies to be detected by standard HIV tests. During the window period, a person may be tested HIV-negative despite having been infected. 2. Not real-time: An individual may be infected after testing negative and still can show a negative result after infection.

The PEFRs can be limited by setting Dapp usage rules. The following analysis mainly shows that even in the presence of PEFRs, the HDV strategy remains overwhelmingly superior: it significantly reduce the potential sources of infection at population level and the probability of exposure to sources of infection at individual level. No strategy is perfect (the discomfort makes condom low acceptability; 90-90-90 is costly and difficult to achieve and maintain), combining HDV with existing measures allows for more efficient HIV surveillance and prevention.

**1. HDV can significantly reduce the infection sources**

According to the US CDC, 37.6% of the newly diagnosed HIV cases in 2016 were generated by individuals unaware of their infection, the remaining 62.4% were generated by individuals aware of their infection [1]. Normally, very few people have unprotected sex with each other knowing that the other person is HIV-infected, and the data above implies that there are serious cases where individuals who aware of their infections do not disclose their HIV-positive status to their sexual partners. HDV can make most of the existing sources of infection unavailable to HDV vaccinees(users who have Dapp installed and have valid test results), with only PEFRs remaining, and the amount is very small.

As shown in Supplementary 1 Figure S1, Y are the infection sources reduced by HDV, which makes it extremely difficult to have sex with committed HDV vaccinees due to the unavailability of HIV-negative results. Even if a small number of HDV vaccinees (such as bugchaser [2]) voluntarily have sex with Y without HDV and being infected, and then become Z (PEFR). However, due to the restrictions of HDV usage rules, Z will soon change back to Y, and the number of vaccinees who may be infected by Z will be limited by rules (see section 2).


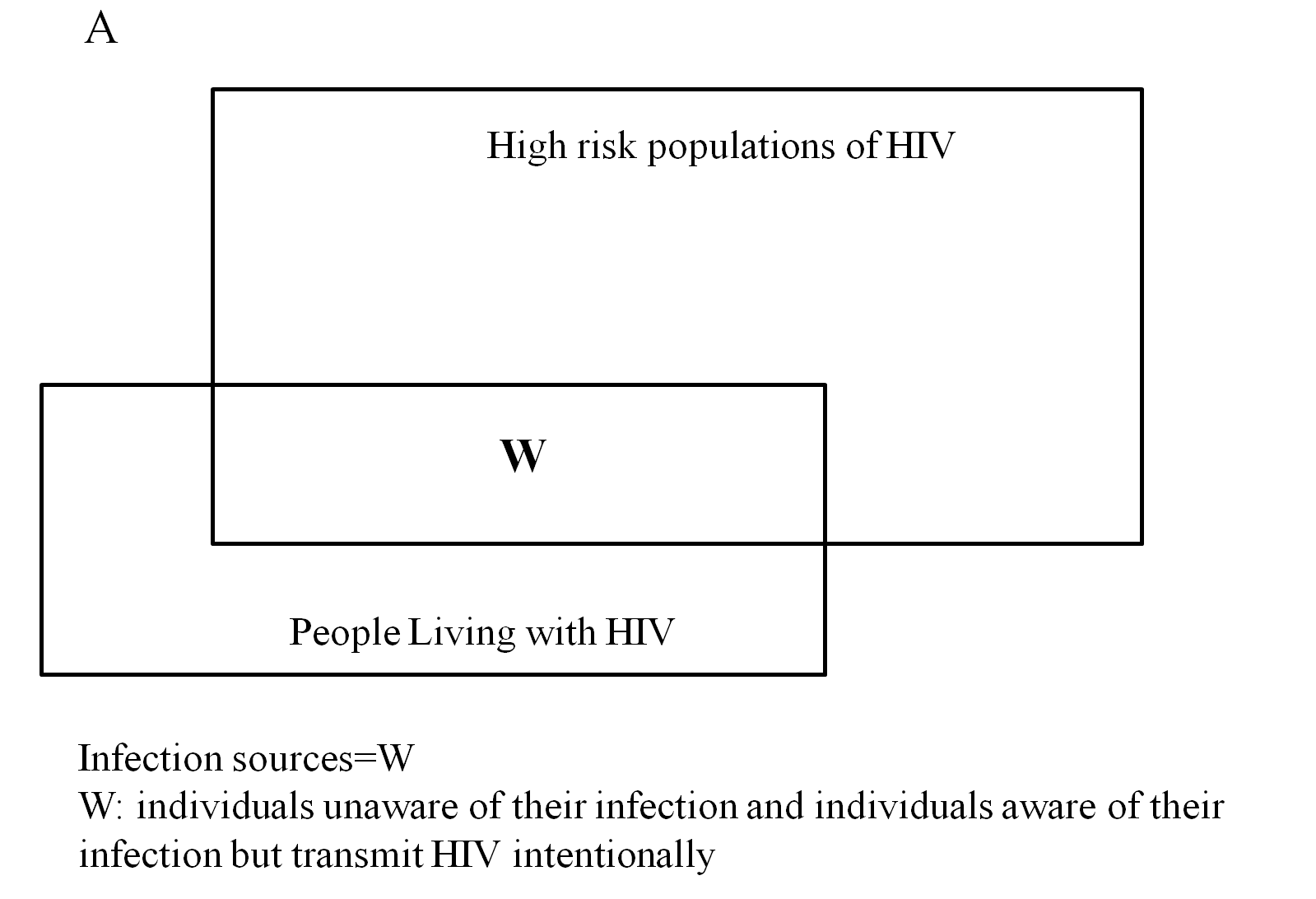


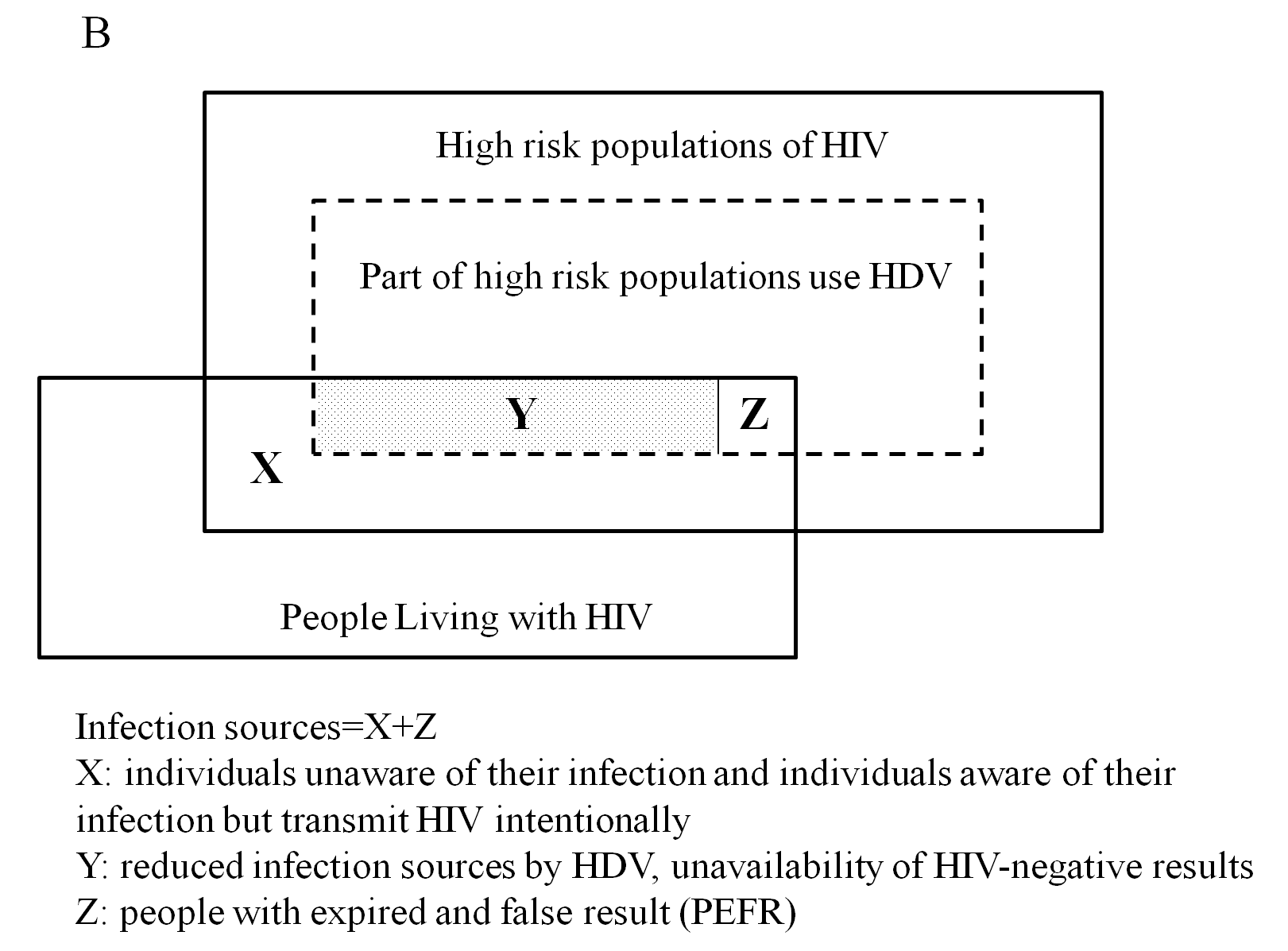

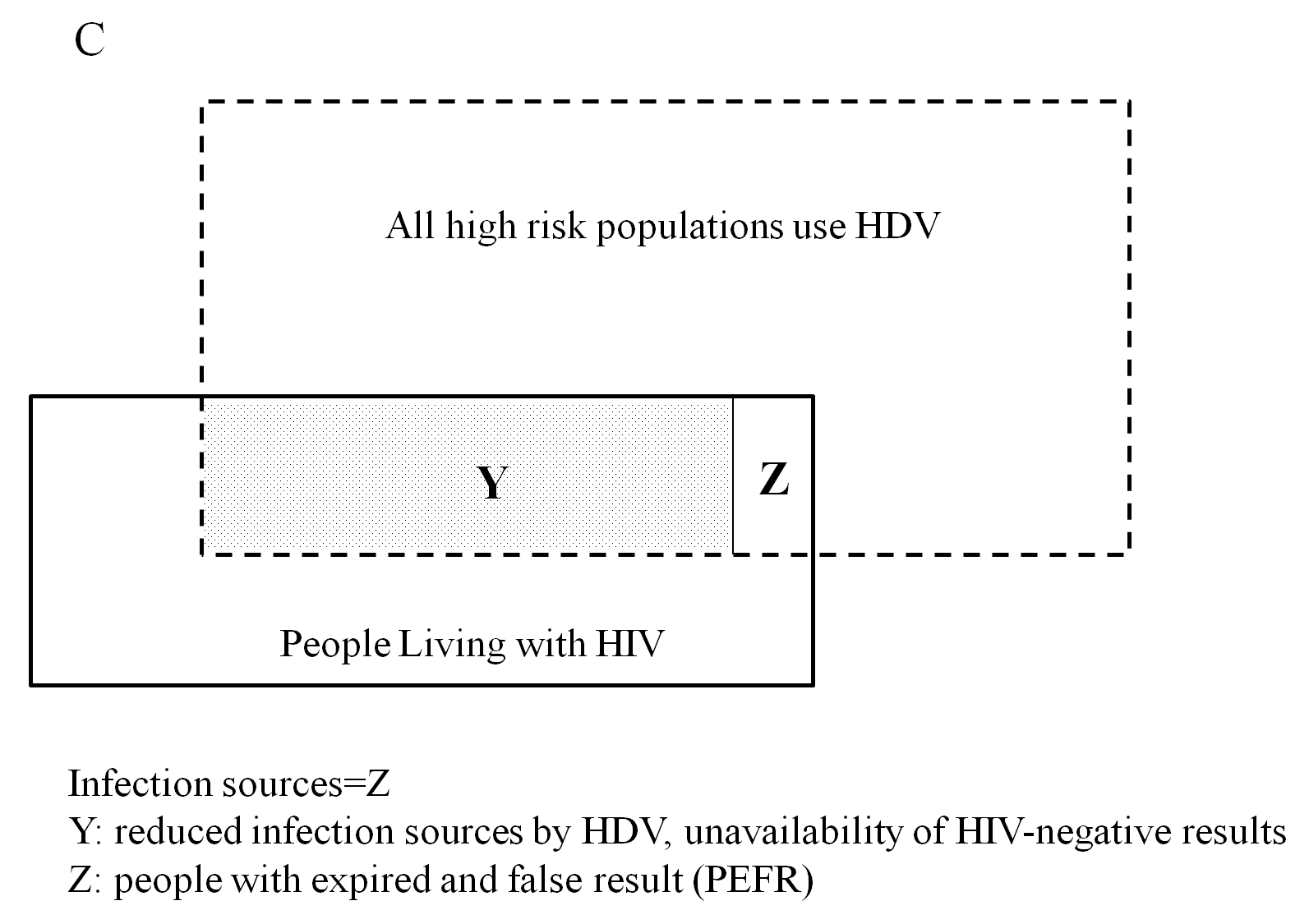


Supplementary 1 Figure S1. Schematic view of infection sources before and after HDV usage. A: Infection sources of HIV in current situation. B: Infection sources of HIV when HDV is used in part of high risk populations. C: Infection sources of HIV when HDV was used in all high risk populations.

For more intuitive, the number of potential sources of infection for the four scenarios was roughly measured and compared (Supplementary 1 Table S1 and Table S2). The raw data used for calculations in the table are from reference [3]. For example, the estimate of People Living with HIV is 100×10^4^, of which 86% were diagnosed, 64% of the diagnosed were treated, 53% of the treated achieved virally suppressed.

The reality in 31/12/2016 (Group R) is that the number of potential sources of infection is 70.8288×10^4^. If achieving 90-90-90 strategy in 31/12/2016 (Group I), the number is 27.1×10^4^. If HDV is used by 63% of the high risk population in 31/12/2016(Group H (63%)), the number is 26.6×10^4^, approximately equal to Group I. If HDV is used by 100% of the high risk population in 31/12/2016(Group H), the number is 0.4×10^4^.

Although the number of sources of infection in the 63% HDV use case is equal to the number in the ideal 90-90-90 case, common sense would infer that the HIV R0 of the non-vaccinees in Group H (63%) should be significantly lower than the high risk population in the Group I case. Because they don't use HDV, it's likely that the reason for the low frequency of high-risk sex, sexual partner fixation, or the availability of applicable protective measures.

More importantly, the non-vaccinees in Group H (63%) had 65.5 times (26.2/0.4) more sources of contagion than the vaccinees in the same group (see Table S2), and the snowball effect of this overwhelming advantage would be favorable for rolling out HDV to higher usage with much lower difficulty and resource consumption than the 90-90-90. Under the ideal scenario of achieving 90-90-90 and 100% usage of HDV, the former (Group I) contagion sources for the entire community are 67.75 times higher than the latter (Group H).

Supplementary 1 Table S1 Estimation and comparison of the HIV potential infection sources in given scenarios (Group R and I) in US, 2016.

|  |  | Estimated HIV infections | Diagnosed (86%) | Unaware of infection | Treated (64%) | Untreated | Virally suppressed(53%) | Unsuppressed | Potential source of infection |
| --- | --- | --- | --- | --- | --- | --- | --- | --- | --- |
| Group R | Reality in 31/12/2016 | 100 | 86 | 14 | 55.04 | 30.96 | 29.1712 | 25.8688 | 70.8288 |
|  |  | Estimated HIV infections | Diagnosed (90%) | Unaware of infection | Treated (90%) | Untreated | Virally suppressed(90%) | Unsuppressed | Potential source of infection |
| Group I | Ideal 90-90-90 in 31/12/2016 | 100 | 90 | 10 | 81 | 9 | 72.9 | 8.1 | 27.1 |

Note: Raw data used for calculation were from reference [3]. All numbers are needed to multiply 10,000.

Supplementary 1 Table S2 Estimation and comparison of the HIV potential infection sources in given scenarios (Group H(63%) and H) in US, 2016.

|  |  | Potential source of infection | Remarks | |
| --- | --- | --- | --- | --- |
| Group H(63%) | HDV were used by 63% HSP^&^ in 31/12/2016 | 26.6 | Potential source of infection for Vaccinees in HDV: | <0.4^#^ |
|  |  |  | Potential source of infection for Non-Vaccinees: 70.8288*(1-63%)= | 26.2 |
| Group H | HDV were used by 100% HSP^&^ in 31/12/2016 | 0.4^#^ | Supposing that the restart procedure(see section 2.3) conducted 30 days ago.  Only the new HIV infections within 30 days(Being infected during 2016.12.1-2016.12.31) can get a HIV-negative result. | |
|  |  |  |  |  |

^#^: There are estimated 46700 newly dignosed HIV in 2016(include 14% unknow), and number is steady in 2014-2018.
So, 3892(=4000) were infected per month in 2016.
^&^: High Risk Population
Note: Raw data used for calculation were from reference [3]. All numbers are needed to multiply 10,000.

**2. Control the remaining sources of infection (PEFRs) by rules**

Z(PEFR) can be controlled by setting the rules for the use of Dapp in HDV. The rules are enforced by computer code and programs so that vaccinees can't break them. Under the HDV rules, the PEFRs are diminishable and controllable. The usage rules of Dapp could include but are not limited to the following.

2.1 Basic setting: The results of the vaccinee can only be viewed after another vaccinee has scanned his or her QR code. This ensures that viewing of the results can only occur between vaccinees.

2.2 Individual-level rules.

A variety of rules can be used to restrict the number of times a result can be viewed and to increase the number of user tests. The rigor of the rules can be adjusted to suit different circumstances and can be used individually or in combination. The general principle is that the more you use it, the more it needs to be tested.

2.2.1 Multiple tests (N tests, 20 days apart between each test) are required before first use. N is used to adjust the cost of becoming a vaccinee, limiting multiple accounts per capita.

2.2.2 Test results can only be scanned by M users (M depend on how many times the vaccinee being tested, meanwhile M≤5 the highest R0 of HIV) and are valid for 3 months. This limits the maximum number of people one Z(PEFR) can infect.

2.2.3 Monitoring the use frequency of accounts, prohibiting its use for 10 days after P times/month, and virus load test (high cost) is required to reopen the account.

2.3 Population-level rules.

A Restart procedure is periodically implemented to remove PEFRs.

The user is informed in advance of the implementation of the Restart procedure, all accounts are deactivated for 20 days, all results are not displayed, user need a new test and upload to enable the account and restore the original results, each Restart procedure will filter out some of the PEFRs, after multiple Restart, the number of PEFRs will be limited to a minimum.

Regardless of how the rules change, it's only a matter of how many times detection is needed for the user, the high risk population is easy to understand and can accept, and itself has a need for test after the high risk sexual behavior, and HDV simply brings that need forward. These rules limit the abuse of HDV, while ensuring the credibility of the results.

An analogy with biological vaccines allows for a more colloquial explanation, with multiple tests similar to the four-shot vaccination process for the rabies vaccine, and the Restart procedure is like a seasonal vaccination against influenza. The Dapp rules are easily and flexibly adjusted at a very low cost, a feature that also gives digital vaccines an advantage over biological vaccines.

It is important to note that the more restrictions on use, the more convenience of use will be impaired and the fewer people will use it, which needs to be adjusted in practice.

**3. User education and pre-use warnings**

As mentioned above, the number of potential infection sources of non-vaccinees in Group H (63%) is 65.5 time to the remaining vaccinees population. These or more precise data make it easy for users to understand the following: 1. The PESRs are few but will always be present. 2. But the probability of encountering a potential infection source for vaccinees is extremely low compared to non-vaccinees.

There are multiple warnings when the user installs and uses the Dapp: 1. this test result only indicates HIV-negative status as of XX (date) and does not guarantee current infection status. Beware of PEFR, other measures (condoms, microbial agents, PreP and PEP, etc.) can ensure that you are safer. 2. Once you have used HDV, keep sexual activity to other vaccinees or people who aren’t high risk population of HIV. A temporary sexual partner who does not have an HDV is asked to use it immediately, and if he insists on not using it, vaccinees could take other options to protect themselves.

HDV is not a stand-alone measure as an integrated response, and its promotion will also contribute to the promotion of other measures (especially expanded testing), people at risk of HIV will make their own choices based on their circumstances, and the people who love them most are actually themselves.

As with COVID19 prevention, the government has informed the population of the risks and prevention measures. So the population can perfectly avoid infection through extreme hard isolation, but there are still people who get infected from meetings, parties or travel. The prevention and control of infectious diseases is therefore trade-offs between the elements of life, such as freedom, convenience and comfort, and health for the individual.

Reference:

[1] Li Z, Purcell DW, Sansom SL, Hayes D, Hall HI. Vital Signs: HIV Transmission Along the Continuum of Care — United States, 2016. *MMWR Morb Mortal Wkly Rep*  (2019) 68: 267–272. DOI: <http://dx.doi.org/10.15585/mmwr.mm6811e1>

[2] Klein, H. (2014). Generationing, stealthing, and gift giving: the intentional transmission of HIV by HIV-positive men to their HIV-negative sex partners. *Health Psychol Res* (2014) 2(3):1582. doi: 10.4081/hpr.2014.1582

[3] Centers for Disease Control and Prevention. Monitoring selected national HIV prevention and care objectives by using HIV surveillance data—United States and 6 dependent areas, 2017. HIV Surveillance Supplemental Report (2019) 24(3).
